# Hospital Progress and Finance

**Published:** 1924-07

**Authors:** 


### HOSPITAL PROGRESS AND FINANCE.

### LORD KNUTSFORD AND HIS CRITICS.

The protest aroused by Lord Knutsford's recent remarks upon Poor Law Infirmaries has led him to reply to some of his critics. He maintained his attitude, and his general answer to the denial that Poor Law Infirmaries are mostly disliked by the sick is this question: "If people did not hate the Poor Law Infirmaries, is it not strange that we should have a waiting list of 1,000, while across the road in Whitechapel Poor Law Institution there were 200 empty beds, and no applicants for admission?" No Board of Guardians seems to have thought of remarking that, whatever be the condition of the infirmaries, people have a reason for shunning them so long as they are penalised, even if they are paying patients, for entering these institutions. The rights of citizenship may not be very valuable, but to be deprived of them by law, to become technically a pauper, is enough in itself to cast odium on institutions the benefits of which can be obtained only at this price. The pauper taint was once both legal and personal. The personal disability has been reduced or has vanished in the up-to-date infirmaries, but the law still makes outcasts of their patients, and no amount of administrative improvement can disguise a fact so objectionable.

### THE ROCKEFELLER GIFT COMPLETED.

The large gathering at Lansdowne House to meet the Duke and Duchess of York, Prince Henry, Prince George, Princess Marie Louise, and the Committee of the Reckefeller Foundation Gift British Endowment Appeal, was gratified to learn that almost the whole of the £180,000 needed to endow sixty beds at the new obstetric hospital at University College Hospital has been raised. Lord Burnham, the treasurer, stated that the cost of raising the money was not expected to exceed ½ per cent., for the sums received when placed on deposit had provided in interest almost enough to pay all expenses. The gift of £1,205,000, made in 1920 by Mr. John Rockefeller, senr., to form an advanced school for medical education and research in London, is thus supplemented, so that all the cost and endowment has been provided, in which that of these sixty beds fell to our country's share. At the banquet and ball at Lansdowne House, which was lent by Mr. Selfridge, selected representatives of the different donors of beds were received by the Royal visitors. The appeal was made on May 7, and on the date of the gathering, May 30, only three beds remained to be endowed.

### LOCAL COMMITTEES AND HOSPITAL BEDS.

The Local Voluntary Hospitals Committees, in pursuance of the inquiry into the additional number of beds required throughout the country, are attempting to estimate the exact number of persons seeking treatment by eliminating duplicate entries at different hospitals, by specifying whether convalescent or hospital beds are needed in their area, and how far the former are provided independently of the hospitals. The Committees are also asked to state the extent of any plan for using vacant beds in Poor Law Infirmaries, and how far vacant beds in hospitals can be used, for example, by the transfer of patients; to ascertain the views of medical officers of health, and to make their own suggestions for increasing local hospital co-operation. It is not a little remarkable that the Government grant should have been the means of stimulating hospital co-operation, and that the organisation thus created should have found continuous and useful work to do after its initial task in distributing the grant was completed.

### THE LIMITS OF VOLUNTARYISM?

At the recent luncheon at the Mansion House, when £4,000 was raised on behalf of the appeal for £30,000 to extend the City of London Hospital (Victoria Park) for Diseases of the Chest, Sir A. Kaye Butterworth, the chairman, said that if the money were found there would be no similar appeals for many years. The object is to complete a new surgical block, to extend the research department, and to furnish the sanatorium at Saunderton, Buckinghamshire. The plan originated in 1914 but was postponed by the war, and as the hospital has contrived to pay its way, the appeal is all the more encouraging. The Duke of Connaught, who presided at the luncheon, has been president for over fifty years, and it is one of the four hospitals with which he is officially connected. The sum required is relatively small, and we particularly wish the appeal success, for the chairman believes that even those most opposed to the principle of State management are now inclined to favour public control of hospitals. We believe this to be due not to dislike of the voluntary principle but to fear that the limits of the voluntary system of raising money have been reached. Every successful appeal, therefore, helps to silence a disquieting practical argument.

### NO MORE BUILDING IN SHEFFIELD.

Mr. Humberstone, president of the Sheffield and District Association of Hospital Contributors, has announced that the four Sheffield hospitals have agreed to erect no more hospital buildings within the area of the city. He added that negotiations were in progress for the purchase of a site, which, if acquired, would enable them to transfer the city hospitals one by one to the new position. The well-known penny in the pound scheme has helped to make this possible, and Mr. Humberstone suggests that the Approved Societies should be approached in the hope of getting more generous financial help from them for the hospitals generally. Mr. Humberstone's speech, however, is chiefly remarkable for the decision it records to build no more hospitals within the city. Such a decision was bound to come, but Sheffield has been the first to make it, and it may well mark a date in the evolution of hospital planning. Towns, however, continue to grow, and all sites purchased outside their present boundaries must keep an eye on the future, and should secure an area much larger than their immediate needs require to protect them against future encroachments. True, we shall not always be content with the smoke-polluted cities of to-day; but even so an absence of neighbouring buildings will remain desirable.

### THE BRADFORD INFIRMARY SITE.

The Bradford City Council has referred back the proposal of the finance committee to offer £75,000 to the governors of the Bradford Royal Infirmary for the purchase of the infirmary site and buildings in Westgate. Were the purchase made the infirmary board would use the money toward the erection of a new hospital at Daisy Hill. The proposal originated ten years ago, but came to nothing through the war, and some of those on the City Council who were then in its favour are now opposed to the plans. They think that the price asked for the infirmary is excessive, and that the hospital needs of the city have been met in the meanwhile through the municipal hospital of St. Luke's. Thus this institution, around which there has been a good deal of controversy at different times, seems likely to prevent the original proposal from being adapted. If so, the difficulties in the way of the new hospital at Daisy Hill will be much increased. Unfortunate as this is, it is still more regrettable that the voluntary and municipal hospital of the city should be regarded as rivals, and the future of the Royal Infirmary become the prey of party feeling.

### THE SUNLIGHT LEAGUE.

The interest aroused by the sunlight treatment at Leysin, Switzerland, carried on under Dr. A. Rollier, who has lately visited England, has resulted, among other things, in the establishment of a Sunlight League. Dr. C. W. Saleeby, appropriately enough, is the chairman of the council, and Dr. Rollier its vice-president. The Duke of Sutherland and Dr. T. A. Palm are the presidents, and the latter has the distinction of having observed the beneficial effects of sunlight many years ago on his return from Japan, where he was nine years a medical missionary. The papers on the subject that he contributed to the *Practitioner* in the autumn of 1890 are now appraised at their true value. The National Institute for Medical Research at Hampstead is following his proposal for the recording of sunshine in smoky cities no less than at health resorts, and the Sunlight League is now starting to educate public opinion against smoke and dark streets and in the value of sun-baths, translucent clothing, and so forth. Further particulars can be had from the hon. secretary, Miss May Scanlan, at the temporary office, 20, Park Crescent, W. The work at Leysin has attained this year its twenty-first birthday, but how long the public knowledge of its value has been in growing!

### AREAS AND CONTRIBUTIONS.

One of the difficulties of the present time is to control the overlapping that is alleged to occur in the matter of hospital appeals. We are told not merely that different hospitals compete with one another for subscriptions, but that some hospitals raise money from areas remote from themselves, and in certain places a desire has been expressed to define each hospital's area. The result of inquiries has taught some useful lessons, if not those that the instigators expected. It is found, for instance, that though appeals might be better arranged than they often are at present, the fact that different hospitals render different services makes it practically impossible to define the area in which they should be allowed to issue appeals. Patients for special hospitals may come from all over a district, and the large hospitals are like special hospitals in that they can treat certain cases better than institutions less well equipped. Consequently the attempt to define geographical areas will have to be abandoned. The solution seems to be, where possible, the establishment of a joint hospital fund, largely maintained by a contributory scheme, the proceeds of which are proportionally divided.

### GLASGOW'S NEW HOSPITAL.

The new buildings of the Glasgow Hospital for Diseases of the Ear, Nose and Throat, near St. Vincent Street, are making progress, and when complete will give the hospital forty-two beds, which is three times the present number. Lord Weir's Fund has now passed £36,000, and the total available for the construction of the new hospital is £51,000. The present accommodation is overcrowded and inadequate. The waiting-list is long, and it is much hoped that the fund will grow sufficiently during the summer to enable the new buildings to be opened free of debt.

### A LATE RECRUIT.

Lincoln is the latest hospital centre to adopt a contributory scheme, which is badly needed there to reduce the debt and to balance the expenditure. An income of £20,000 a year is required to maintain the Lincoln County Hospital, and this attempt to secure it has been made none too soon. The city and county, however, have begun to take the scheme in hand, and all experience shows that the difficulty is at the start and not in the extension of weekly contributions. We trust, therefore that the organisation will be vigorously pressed forward, for the growing debt, now amounting to £6,000, can be met in no other way. Indeed, an institution in the condition of the Lincoln County Hospital, which is without such a scheme, deliberately places itself at a disadvantage.

### TESTATORS AND LOCAL COMMITTEES.

The latest Local Committee to appeal in its own name for money is that of the East and West Ridings of Yorkshire, of which Lord Lascelles is chairman. In a statement of its aims and work, we read "it is the intention of the Yorkshire Committee to appeal for support similar to that accorded by the public" to King Edward's Hospital Fund. As the fund, of course, acts as the Local Committee for London, there is a precedent which has probably encouraged the idea, for hitherto all attempts to found in the provinces funds similar to the King's Fund have proved abortive. On the other hand, probably no money is subscribed expressly to the Local Voluntary Hospital Committee for London, and it seems worth while to ask if, in their capacity as funds inviting subscriptions, the Local Committees are very fortunately named.

